# Supplementary material for: Identifying repeat domains in large genomes
Source: Genome Biol. 2006 Jan 31;7(1):R7. doi: 10.1186/gb-2006-7-1-r7 (PMC1431705; doi:10.1186/gb-2006-7-1-r7)
Supplement: Additional File 1 — A zipped file of browsable HTML files with a complete list of the connected components in the repeat domain graph of human Repbase. [file gb-2006-7-1-r7-S1.gz › html/subgraphs/7222.pdf]

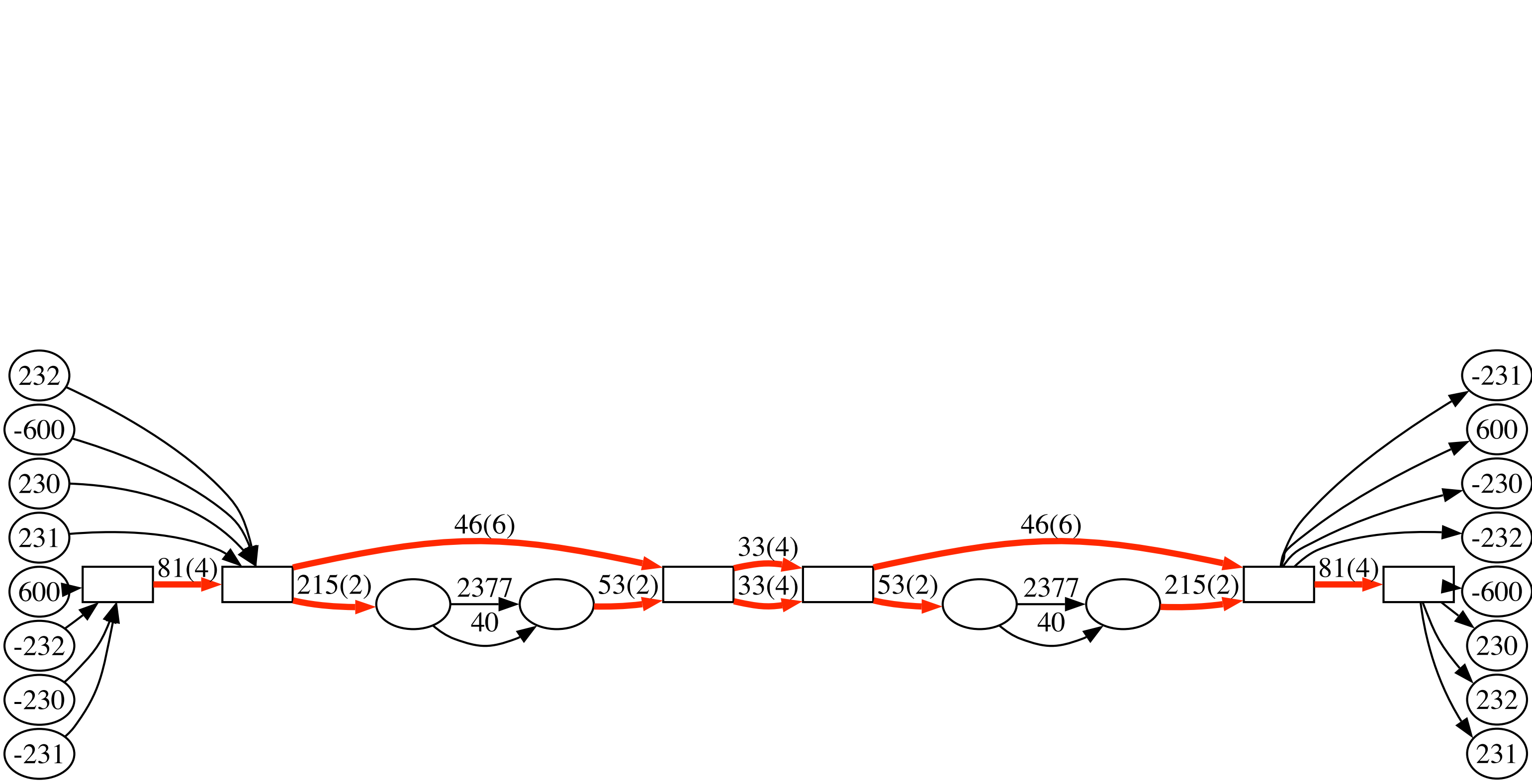

232
-600
230
231
600
-232
-230
-231
81(4)
46(6)
215(2)
2377
40
53(2)
33(4)
33(4)
46(6)
53(2)
2377
40
215(2)
81(4)
-231
600
-230
-232
-600
230
232
231
